# Evaluating the Synergistic Neutralizing Effect of Anti-Botulinum Oligoclonal Antibody Preparations

**DOI:** 10.1371/journal.pone.0087089

**Published:** 2014-01-27

**Authors:** Eran Diamant, Bat-El Lachmi, Adi Keren, Ada Barnea, Hadar Marcus, Shoshana Cohen, Alon Ben David, Ran Zichel

**Affiliations:** 1 Department of Biotechnology, Israel Institute for Biological Research, Ness Ziona, Israel; 2 Department of Infectious Diseases, Israel Institute for Biological Research, Ness Ziona, Israel; Naval Research Laboratory, United States of America

## Abstract

Botulinum neurotoxins (BoNT) are considered some of the most lethal known substances. There are seven botulinum serotypes, of which types A, B and E cause most human botulism cases. Anti-botulinum polyclonal antibodies (PAbs) are currently used for both detection and treatment of the disease. However, significant improvements in immunoassay specificity and treatment safety may be made using monoclonal antibodies (MAbs). In this study, we present an approach for the simultaneous generation of highly specific and neutralizing MAbs against botulinum serotypes A, B, and E in a single process. The approach relies on immunization of mice with a trivalent mixture of recombinant C-terminal fragment (Hc) of each of the three neurotoxins, followed by a parallel differential robotic hybridoma screening. This strategy enabled the cloning of seven to nine MAbs against each serotype. The majority of the MAbs possessed higher anti-botulinum ELISA titers than anti-botulinum PAbs and had up to five orders of magnitude greater specificity. When tested for their potency in mice, neutralizing MAbs were obtained for all three serotypes and protected against toxin doses of 10 MsLD_50_–500 MsLD_50_. A strong synergistic effect of up to 400-fold enhancement in the neutralizing activity was observed when serotype-specific MAbs were combined. Furthermore, the highly protective oligoclonal combinations were as potent as a horse-derived PAb pharmaceutical preparation. Interestingly, MAbs that failed to demonstrate individual neutralizing activity were observed to make a significant contribution to the synergistic effect in the oligoclonal preparation. Together, the trivalent immunization strategy and differential screening approach enabled us to generate highly specific MAbs against each of the A, B, and E BoNTs. These new MAbs may possess diagnostic and therapeutic potential.

## Introduction

Botulinum neurotoxins (BoNT), produced by *Clostridium botulinum* strains, are considered the most lethal toxins known, with an estimated human median lethal dose (HLD_50_) of 1 ng/kg body weight [1], [2]. Seven immunological BoNT serotypes (A–G) are known, of which types A, B, E, and rarely F are responsible for most cases of human botulism [3].

Botulinum toxins are synthesized as large protein complexes consisting of a neurotoxin, non-toxic hemaglutinins (HA), and non-toxic non-hemaglutinins (NTNH) [4]. The active form of the neurotoxin consists of 100,000 (heavy chain) and 50,000 (light chain) Dalton polypeptide chains, which are joined by a disulfide bridge [5]. The C-terminal half (∼50 kDa) of the heavy chain (Hc) is the receptor binding domain while the N-terminal half (Hn) is the translocation domain of the neurotoxin. The catalytic domain is a zinc-endopeptidase confined to the light chain (L) [6].

The alignment of the different BoNT serotypes reveals that the surface residues of Hc vary dramatically among these toxins [6]. Moreover, although Hc itself is non-toxic, most of the neutralizing epitopes have been mapped to the Hc fragment [7]. These characteristics make the Hc fragment a promising vaccine candidate [8], [9], [10] and a target immunogen for the production of highly specific Abs for differential diagnosis of botulinum serotypes.

Due to their extreme potency and lethality, ease of production and transport, and need for prolonged intensive care [1] BoNTs are the only toxins classified by the CDC as category A agents. Therefore, early diagnosis and treatment are of high importance in botulism patients. The standard treatment for botulism relies on polyclonal antibody (PAb)-based antitoxin therapy, together with supportive care, i.e., mechanical ventilation [11]. Current pharmaceutical anti-botulinum drugs for adults are produced from hyperimmune horses and have significant side effects, including hypersensitivity reactions such as serum sickness and anaphylaxis [12], [13].

For diagnostic purposes, the mouse bioassay is used as a confirmatory test to demonstrate the presence of toxin in suspected specimens [11]. This assay is very sensitive, but it involves the use of live animals and is time consuming. Furthermore, additional tests with neutralizing antibodies must be conducted to determine the toxin serotype. As a result, there has been tremendous progress in the development of alternative tests, including mass spectrometry based assays and various sensitive immunoassay formats [14]. Nevertheless, the differential *in vitro* diagnosis of botulinum toxins is challenging mainly due to the demand for both high affinity and highly specific antibodies that will detect the extremely low serum concentration of these highly potent toxins.

Monoclonal antibodies (MAbs) with high specificity and defined properties have the potential to address some PAb limitations associated with the diagnosis and treatment of botulism. Substantial advances in screening procedures have been applied since the development of hybridoma technology. For instance, the use of less time and labor consuming screening technologies such as robotic-based assays, allows handling a significantly higher number of hybridomas in a single process. [15].

The use of MAbs should prove very beneficial as highly specific agents in immunoassays. Indeed, many anti-BoNT MAbs have been integrated in detection immunoassays. The ability to sensitively detect different BoNTs and BoNT complexes in relevant specimens such as food products and bodily fluids was demonstrated by various techniques in which MAbs were used (as capture Abs). Examples of these techniques include the following: amplified ELISA [16], electrochemiluminescence (ECL) [17], immunoaffinity chromatographic column tests [18], colorimetric s-ELISA with both capturing and detecting MAbs [19], functional dual-coated (FDC) methods [20], and others.

MAbs may become highly advantageous for use as potential therapeutic drugs. Numerous Mabs have been purified and characterized for their protective efficacy against different toxins [21]. Compared to conventional chemical therapies MAbs show favorable pharmacokinetics and pharmacotoxicity and have fewer side effects due to their high specificity and affinity to the disease target [15]. Neutralizing MAb-based preparations may be safer alternatives in the future as the humanization of well-defined mouse MAb sequences can now be performed routinely. As a result, the immunogenicity of the new antibodies should be substantially reduced. The first pharmaceutical attempts in the botulinum countermeasure field focused on anti-BoNT/A, B, and E MAb-based antitoxin drugs [13], [22].

In this study, we immunized mice with a trivalent mixture of the recombinant Hc of botulinum neurotoxins A, B, and E, followed by simultaneous differential robotic hybridoma screening. This approach was used to develop highly specific monoclonal antibodies against A, B and E botulinum neurotoxins. Seven to nine MAbs against each serotype were cloned, characterized, and found to have very high anti-toxin titers. Individual neutralizing MAbs against each toxin serotype were obtained and high synergistic neutralizing effects were demonstrated for different combinations of MAbs. Our approach proved to be useful in generating highly specific MAbs to BoNTs with diagnostic and therapeutic potential.

## Materials and Methods

### Ethic Statement

All animal experiments were performed in strict accordance with the Israeli Law and were approved by the Ethics Committee for animal experiments at the Israel Institute for Biological Research (permit no: M-43-2009 and M-09-2012). All efforts were made to minimize suffering. During the survival studies, loss of righting reflex was used as the humane end point of the experiment. Mice were monitored three times a day for their condition and for the occurrence of end point. Mice that presented loss of righting reflex were humanely euthanized and sacrificed.

### Bacteria, Toxins, and Toxoids

*Clostridium botulinum* A, B and E strains were obtained from the IIBR collection (A198, B592 and E450, respectively). Sequence analysis revealed compliance of the neurotoxin genes with serotypes 62A (Accession Number M30196), Danish (Accession Number M81186) and NCTC11219 (Accession Number X62683) for *Clostridium botulinum* types A, B and E, respectively [23], [24], [25].

*Clostridium botulinum* A, B and E toxins were prepared from concentrated supernatants of cultures grown for 6 days in anaerobic culture tubes.

To prepare toxoids, the purified A, B, and E toxin complexes were dialyzed against 0.14%–0.2% formalin at 35°C for 2–4 weeks.

### Cell Culture Media

Solutions and reagents were from Biological Industries (Beit Haemek, Israel) unless otherwise stated.

Hybridoma medium (HyMed) included RPMI with high glucose 4.5 g/L, 10% FBS heat inactivated sera, 2 mM L-glutamine, 10 mM HEPES, 1 mM sodium pyruvate, 1.5 g/L sodium bicarbonate, Minimal Essential Amino Acid Solution 1∶100, and 0.1 mg/ml Gentamycine sulfate.

HAT selection medium included 20 ml of Hypoxantine, Aminopterine, Tymidine cocktail in 1 L of HyMed. HT selection medium included 20 ml of Hypoxantine, Tymidine cocktail in 1 L of HyMed.

Enriched DMEM solution (EnDMS) contained DMEM with 4.5 g/L D-glucose, 2 mM L-glutamine, 10% FBS heat inactivated sera, and 0.1 mg/ml gentamycin sulphate.

All secondary antibodies, alkaline phosphatase (AP) or HRP conjugated secondary antibodies, were purchased from Jackson ImmunoResearch Laboratories Inc., USA, unless otherwise mentioned.

### Immunization of Mice

**Figure 1:**
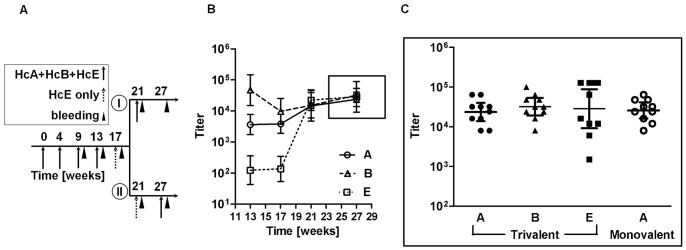
Anti-serotype A, B, and E ELISA titers in mice immunized by a trivalent protocol. **A.** Immunization scheme **-** Ten female Balb/c mice were immunized s.c. with a trivalent vaccine containing 5 µg of each of the three Hc fragments, HcA, HcB, and HcE, in CFA (first injection) or IFA (second and third injections). Booster immunizations of trivalent or monovalent (HcE alone) vaccines were given i.m. at 4-week intervals. After three boosters, the immunization regimen was split into two (I and II). Then, two groups of five mice were differentially injected according to their ELISA titers. Mice were bled for titer analysis ten days after each immunization. **B**. ELISA titer development from week 13 through week 27 for the two combined protocols. As no significant difference in ELISA titer was observed between the two immunization groups (I and II), the data represent the geometric mean of all 10 mice with 95% confidence levels. **C**. Final titers of anti-serotype A, B, and E (at week 27) in the trivalent combined protocols compared to titers of anti-serotype A in the HcA monovalent control. Geometric means with 95% confidence levels are presented for each of the 10 animal groups (trivalent protocol: anti-A –23,600, anti-B –32,000, anti-E –28,500; monovalent protocol: anti-A –25,900).

Female BALB/c mice (Charles River, UK) were immunized subcutaneously (S.C.) with a purified trivalent vaccine preparation containing 5 µg of HcA, HcB, and HcE fragments [8] in 100 µl of phosphate-buffered saline (PBS) emulsified with 100 µl of Complete Freund’s Adjuvant (CFA, Sigma Aldrich, USA). Mice received two boosts of trivalent vaccine emulsified with Incomplete Freund’s Adjuvant (IFA, Sigma) at weeks 4 and 8. Then, the mice received soluble boosts of trivalent or monovalent HcE vaccine preparations at 4-week intervals, as shown in Figure 1A. Ten days after each immunization, sera samples were collected for titer analysis.

### Fusion Procedure

Cell fusion was performed according to the classical method [26], [27]. Following the last I.P. injection, the mouse with the highest anti-A, anti-B, and anti-E serum titers was boosted with an intravenous (I.V.) injection of a soluble trivalent (10 µg HcA +10 µg HcB +10 µg HcE) vaccine preparation. Three days after the I.V. injection, the mouse was euthanized and its splenocytes were fused with exponentially growing myeloma cells using 50% polyethylene glycol (PEG) 1500 in EnDMS. Following cell fusion, the cells were suspended in HAT selection medium together with splenocytes from a naïve non-immunized mouse that was used as a feeder layer. The cells were dispensed into 96-well tissue culture plates (Greiner, Austria) and incubated for 5–10 days at 37°C in 5% CO_2_ before screening for secreted antibodies.

### Robotic Screening

Five days post fusion, the hybridoma culture plates were simultaneously screened at 2-day intervals for anti-A, anti-B, and anti-E antibody secretion by automated ELISA using two robots.

MaxiSorp ELISA plates (Nunc, Denmark) were coated with 10 µg/well of toxoid A, B, or E diluted in coating buffer (50 mM Na_2_CO_3_, pH 9.6) and incubated overnight at 4°C. The plates were then blocked for 1 h at 37°C with TSTA buffer (50 mM Tris, 0.9% NaCl, 0.05% Tween 20, 2% bovine serum albumin; 250 µl/well). The supernatants from each well of the hybridoma cell culture plate were distributed into three wells of different antigen-coated ELISA plates using 50 µl per well. These plates corresponded to anti-A, B, and E assays. The plates were then incubated for 1 h at 37°C. Next, twenty-four ELISA plates and the relevant buffers were concurrently placed in two robotic machines (GENESIS Robotic Microplate Processor (RMP) 200/150, TECAN, Switzerland). There were 12 plates per robot, and the sequence of events followed the designed programs. Twice each day, the negative and positive controls were added, and the plates were incubated for 1 h at 37°C. The plates were then washed three times with saline containing 0.05% (W/W) Tween 20 (washing buffer, WB). Horseradish peroxidase (HRP)- or alkaline phosphatase (ALP)-conjugated goat anti-mouse IgG was diluted 1∶2,000 or 1∶1,000, respectively, in TSTA (for anti-A or anti-B and -E assays). The antibodies were added, and the plates were incubated for 1 h at 37°C. The plates were washed three times with WB, and TMB (Sigma) or pNPP (Sigma) substrate was added for the anti-A or anti-B and -E assays. The plates were incubated for 15 min at 37°C. For the anti-A assays, the enzymatic reaction was stopped with 100 µl/well of 0.5 M H_2_SO_4_. Finally, the optical density was determined at 450 nm (anti-A plates) or 405 nm (anti-B and anti–E plates) in a SpectraMax 190 spectrophotometer (Molecular Devices, USA).

### Determination of Total IgG Concentration in Ascites Fluids

To evaluate the total mouse IgG concentration in the ascites fluids (AF) samples, ELISA plates (MAxisorp, Nunc) were coated with 120 ng (50 µl) goat anti-mouse IgG, F(ab′)_2_ fragment specific in coating buffer (50 mM Na_2_CO_3_, pH 9.6) and incubated overnight at 4°C. Plates were then washed in PBST buffer and blocked for 1 hr at 37°C with 200 µl per well of 2% (W/V) BSA in Tris-NaCl pH 7.6 (TSTA). Following blocking and washing, plates were incubated with serial dilutions (50 µl, in duplicate) of the AF samples in TSTA (50 µl/well, in duplicate) for 1 hr at 37°C. After washing with PBST, plates were incubated with 30 ng (50 µl) alkaline phosphatase-conjugated donkey anti-mouse IgG diluted in TSTA for 1 hr at 37°C. Finally, plates were washed with PBST, and the color reaction was developed using p-Nitrophenyl substrate (Sigma). Absorbance was measured at 405 nm with Molecular Devices Spectramax M3 reader. The IgG concentration was determined by interpolation from a 1.56–100 ng/ml standard curve (fitted to four-parameter equation) of ChromPure mouse IgG whole molecule, by SoftMax Pro software. IgG concentration of all MAbs is presented in Table S1.

### Establishment of Stable Clones

Hybridoma well volumes were manually completed by the addition of 100 µl of fresh HAT medium. During cell growth, the HAT selection medium was replaced with HT medium, and secreting cells were further grown in HyMed without HT or HAT.

Hybridoma cells from the wells showing positive signals for antibody secretion were cloned twice by limiting dilution. The hybridoma cells were then gradually expanded in HyMed, and 10^7^ cells/ml from 10 cm plates were collected and frozen in HyMed containing 10% dimethyl sulfoxide (DMSO, Sigma).

The subclass of each MAb was determined from hybridoma cell culture supernatants using a mouse monoclonal isotyping kit (MMT1, AbD Serotec, USA) according to the manufacturer’s instructions.

Ascites fluids were obtained from 6- to 8-week-old female BALB/c mice. Mice that were pretreated with Pristane (Sigma) were injected with 5×10^5^ hybridoma cells in PBS. Approximately 10–20 days post injection, ascites fluids and cells were collected. The fluids were frozen at −20°C, and the cells were suspended in HyMed containing 50% FBS and 10% DMSO and frozen in liquid nitrogen.

### Determination of Anti-toxin Titers using Sandwich-ELISA

The ability of MAbs to bind native toxins was determined by sandwich ELISA (s-ELISA). ELISA plates were coated with 0.5 µg/well of Rabbit anti-complex A, B, or E PAbs diluted in coating buffer [8]. The plates were incubated overnight at 4°C. The plates were then washed three times with WB and blocked for 1 h at 37°C with TSTA. The plates were washed once, and then native toxin A, B, or E (2–25 ng/well) was added and the plates were incubated for 1 h at 37°C. The plates were washed four times, and then serial dilutions of MAbs were added and the plates were incubated for 1 h at 37°C. After four washes, HRP-conjugated donkey anti-mouse IgG, diluted 1∶1,000 in TSTA, was added and plates were incubated for 1 h at 37°C. The plates were washed four times, and TMB (Sigma) substrate (50 µl/well) was added. The enzymatic reaction was stopped with 100 µl/well of 0.5 M H_2_SO_4_ after 15 min of incubation at 37°C. Finally, absorbance was measured at 450 nm, and titers were determined as the last dilution with a signal greater than three standard deviations above control naïve sera.

### Assessing Epitope Recognition Groups with Competitive s-ELISA

Distinct epitopes recognized by MAbs of the same anti-serotype binding were assessed with competitive s-ELISA. MAbs were purified using SpinTrap columns (GE Healthcare, Sweden) and conjugated with biotin using EZ-Link Sulfo-NHS-LC -Biotin (Thermo Scientific, USA) according to the manufacturer’s instructions. ELISA plates were coated with 0.5 µg/well of rabbit anti-complex A, B, or E PAbs diluted in coating buffer. The plates were then incubated overnight at 4°C. The plates were then washed three times with WB and blocked for 1 h at 37°C with TSTA. Plates were washed once, and then native toxin A, B, or E (1, 2, 25 ng NT/well, respectively) was added, and then the plates were incubated for 1 h at 37°C. Serial dilutions of purified unlabeled MAbs were mixed with a constant concentration of each of the biotin-labeled MAbs in Deep Well Plate PP 1.2 ml (TreffLab, Switzerland). ELISA plates were washed four times, and competing unlabeled and biotin-labeled MAbs were added, ensuring that all possible combinations of MAb pairs were covered. The plates were incubated for 1 h at 37°C. After four washes, ALP-conjugated streptavidin (SA), diluted 1∶3,000 (in anti-serotype A assay), or HRP-conjugated SA (R&D Systems, USA), diluted 1∶200 (in anti-serotypes B and E assays) was added, and the plates were incubated for 1 h at 37°C. The plates were washed four times, and then pNPP or TMB substrate (Sigma) was added. For the TMB treated plates, the enzymatic reaction was stopped with 100 µl/well of 0.5 M H_2_SO_4_ after 15 min of incubation at 37°C. Finally, absorbance was measured at 405 or 450 nm, and percentages of competition for each MAb pair were determined.

### Neutralization Assay - Variable Toxin Concentrations

MAb ascites were diluted to 1∶100 (∼50 µg/ml IgG) in gelatin buffer (0.2% w/v gelatin in phosphate buffer, pH = 6.4) and incubated with equal volumes of solution containing different concentrations of the relevant toxin (A, B, or E) for 1 h at 25°C. Thereafter, each mixture was injected intraperitoneally (I.P.) to ICR female mice (2 mice, 1 ml per mouse), and survival was monitored for 10 days. MAb was considered neutralizing if 100% survival was achieved.

### Neutralization Assay – Fixed Toxin Concentration

The reference method L+10 from the Pharmacopoeia [28] was used to determine the neutralizing activity of oligoclonal antibody combinations. Briefly, cocktails were made by mixing MAb ascites and diluting the mixture to the final concentration (defined as the dilution of each MAb in the oligoclonal cocktail) to be used in the neutralization assay. Then, 1.2-fold serial dilutions were incubated for 1 h at 25°C with a toxin reference dose previously determined according to the Pharmacopeia. Each dilution was injected I.P. into three mice (1 ml per mouse), and survival was monitored for 10 days. The concentration in International Units (IU/ml) was calculated based on the last dilution in which survival was observed. Anti-BoNT PAbs were used as reference standards.

### Statistical Analysis

ANOVA was used to analyze differences among anti-serotype specific (A, B, and E) serum titers in hyper-immune mice. Differences between anti-A titers following monovalent and trivalent immunization protocols were analyzed with t-tests using GraphPad Prism 5 software, and the results were considered significant when p<0.05.

## Results

### Trivalent Immunization and Preparation of Monoclonal Antibodies Specific to BoNT Serotypes A, B and E

Mice were immunized with a trivalent Hc vaccine (3HcV) containing 5 µg of each of the Hc fragments of botulinum serotype A, B, and E toxins (Figure 1A). Serum titers were determined using a specific ELISA assay in which formalin treated toxin (toxoid) was used as a capture antigen. ELISA assays conducted after three priming immunization with an adjuvant and one soluble 3HcV booster showed significant lower anti-E as compared to anti-A and anti-B titers (Figure 1B, week 13). In an attempt to reach a similar high antibody titer for all three antigens, the protocol was split at week 17 into two immunization regimens, as described in Figure 1A. Similar average anti-serotype A, B, and E titers were measured in the sera of hyperimmune mice at week 21 (Figure 1B). The geometric mean of anti-A, B and E titers after the last immunization was 23,600, 32,000, and 28,500, respectively (n = 10, p = 0.82, Figure 1C).

To confirm that the trivalent immunization protocol was not inferior to a mono-valent protocol, control mice were immunized with HcA alone (Figure 1C). Indeed, no statistically significant differences were observed between the anti-A titers of the trivalent (23,600) and the monovalent (25,900) immunization protocols (n = 10, p = 0.77). This result confirms that antigenic competition does not restrict the potential of the humoral immune response of each Hc antigen. Therefore, the trivalent and monovalent approaches are equally potent.

Spleen cells of the mouse that presented the highest trivalent anti-A/B/E titers were fused with NS1 myeloma cells, as described in materials and methods. The supernatants of hybridoma cells were simultaneously screened using three parallel, serotype A-, B-, and E-specific assays (Figure S1), which were conducted using two robots capable of handling 24 microtiter plates in each session.

Positive hybridoma cells were sub-cloned by limiting dilution to generate nine clones of anti-HcA, seven clones of anti-HcB, and eight clones of anti-HcE specificity. Only one hybridoma line reacted with both type A and type E botulinum (not shown). An isotype analysis indicated that all MAbs consisted of kappa light chains and that the IgG1 isotype was dominant (71%–88%), although IgG2a and IgG2b isotypes were also present.

### Determining Specificity of Generated MAbs

The specificity of the obtained MAbs (summarized in Tables 1, 2, 3) was evaluated by comparing the homologous versus heterologous ELISA titers of ascites fluids against type A, B, and E toxoids (Figure S2). Anti-Hc and anti-complex PAbs were used as controls.

**Table 1:** Titer, isotype, and specificity of anti-BoNT A MAbs.

Antibody		Isotype^a^		ELISA Titer^b^	Specificity^c^	
		H	L		to B	to E
**MAb**	A-1	IgG2a	Igκ	5,120,000	51,200	51,200
	A-2	IgG1	Igκ	2,560,000	25,600	25,600
	A-3	IgG1	Igκ	1,280,000	12,800	12,800
	A-4	IgG1	Igκ	1,280,000	12,800	12,800
	A-5	IgG1	Igκ	1,280,000	1,600	800
	A-6	IgG2a	Igκ	640,000	6,400	6,400
	A-7	IgG1	Igκ	640,000	6,400	6,400
	A-8	IgG1	Igκ	320,000	800	400
	A-9	IgG1	Igκ	80,000	400	400
**PAb**	αHcA			320,000	800	800
	αComplex A			1,280,000	4	256

a Ig isotypes (H = heavy chain, L = light chain) were determined using a commercial kit (AbD Serotec, USA).

b Titers were measured by ELISA using toxoids as capture antigens.

C Specificity was determined by dividing the homologous titer with the heterologous cross-titer. The minimum titer was set at 100.

**Table 2:** Titer, isotype, and specificity of anti-BoNT B MAbs.

Antibody		Isotype^a^		ELISA Titer^b^	Specificity^c^	
		H	L		to A	to E
**MAb**	B-1	IgG1	Igκ	10,240,000	51,200	102,400
	B-2	IgG1	Igκ	10,240,000	12,800	51,200
	B-3	IgG2b	Igκ	10,240,000	3,200	12,800
	B-4	IgG1	Igκ	5,120,000	25,600	51,200
	B-5	IgG1	Igκ	2,560,000	12,800	51,200
	B-6	IgG2b	Igκ	320,000	1,600	6,400
	B-7	IgG1	Igκ	160,000	200	50
**PAb**	αHcB			160,000	50	200
	αComplex B			5,120,000	1	512

a Ig isotypes (H = heavy chain, L = light chain) were determined using a commercial kit (AbD Serotec, USA).

b Titers were measured by ELISA using toxoids as capture antigens.

C Specificity was determined by dividing the homologous titer with the heterologous cross-titer. The minimum titer was set at 100.

**Table 3:** Titer, isotype, and specificity of anti-BoNT E MAbs.

Antibody		Isotype^a^		ELISA Titer^b^	Specificity^c^	
		H	L		to A	to B
**MAb**	E-1	IgG1	Igκ	10,240,000	102,400	51,200
	E-2	IgG2a	Igκ	10,240,000	20,480	51,200
	E-3	IgG1	Igκ	640,000	3,200	3,200
	E-4	IgG1	Igκ	640,000	800	1,600
	E-5	IgG1	Igκ	320,000	1,600	1,600
	E-6	IgG1	Igκ	320,000	640	1,600
	E-7	IgG1	Igκ	320,000	800	800
	E-8	IgG1	Igκ	320,000	50	200
**PAb**	αHcE			960,000	75	300
	αComplex E			1,280,000	32	128

a Ig isotypes (H = heavy chain, L = light chain) were determined using a commercial kit (AbD Serotec, USA).

b Titers were measured by ELISA using toxoids as capture antigens.

C Specificity was determined by dividing the homologous titer with the heterologous cross-titer. The minimum titer was set at 100.

ELISA titers for the majority of anti-serotype A and B MAbs were higher than the anti-complex polyclonal controls. Additionally, two of the anti-serotype E MAbs had anti-botulinum titers that were more than one order of magnitude higher than those of their polyclonal counterpart. The specificity of most of the MAbs was found to be three and five orders of magnitude higher than anti-Hc and anti-complex PAbs, respectively (Tables 1–3). Results and rank of titers were essentially not affected when ELISA titers were normalized to IgG concentration of each MAb ascites sample.

**Figure 2:**
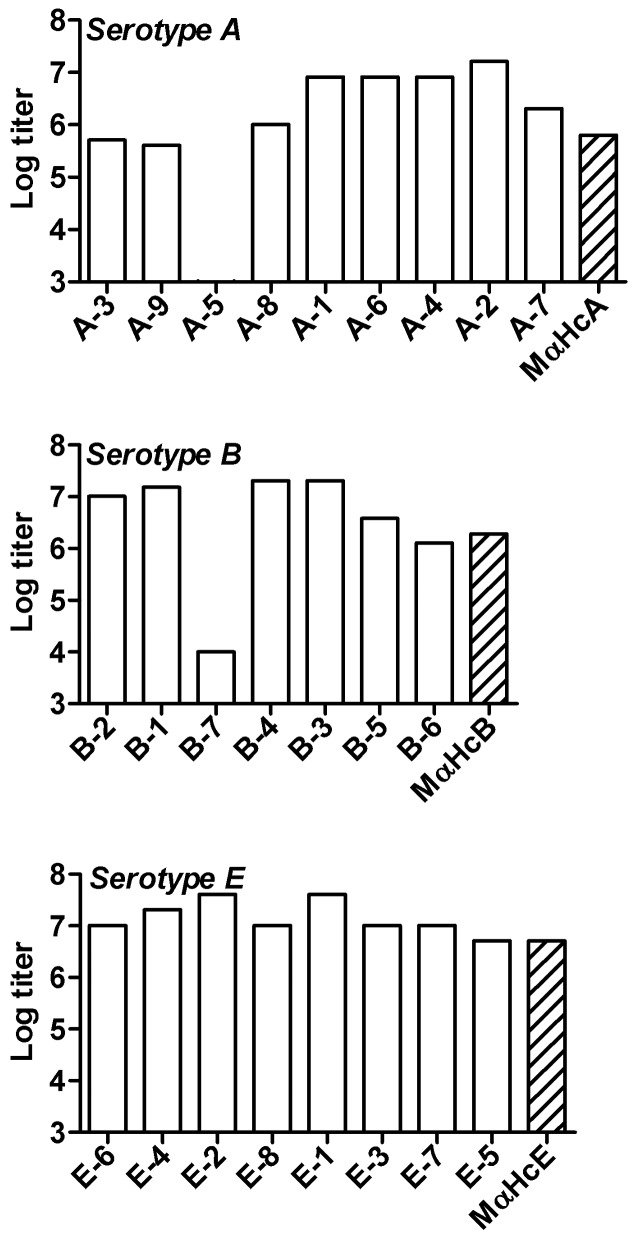
Binding Native toxin. Anti-native BoNT A, B and E titers of MAbs and control PAbs were determined by s-ELISA. Rabbit anti serotype-specific complex A, B, or E PAbs were used to capture toxin, and donkey anti-mouse IgG HRP-conjugate was used to detect bound antibodies. Titers were determined as the last dilution having signal at 450 nm greater than three standard deviations above control naive sera. Striped bars represent mouse anti-Hc PAb controls.

The MAbs were screened and characterized using toxoids as the capture antigen. To determine whether MAbs could bind native toxin, sandwich ELISA (s-ELISA) was established, in which native A, B, or E toxins were captured by anti-complex PAbs. The titers of MAbs to native toxins were determined and compared to the homologous anti-Hc PAbs as controls (Figure 2).

Twenty-three out of twenty-four MAbs bound their homologous native toxin, and most of the MAbs displayed similar or higher titers compared to their PAb counterparts. MAb A-5 (anti-serotype A) was the only MAb that did not recognize its native toxin. These results confirm that screening with toxoid as the capture antigen still allows the generation of MAbs that can strongly bind their homologous native toxins.

### Determination of the Neutralizing Activity

**Figure 3:**
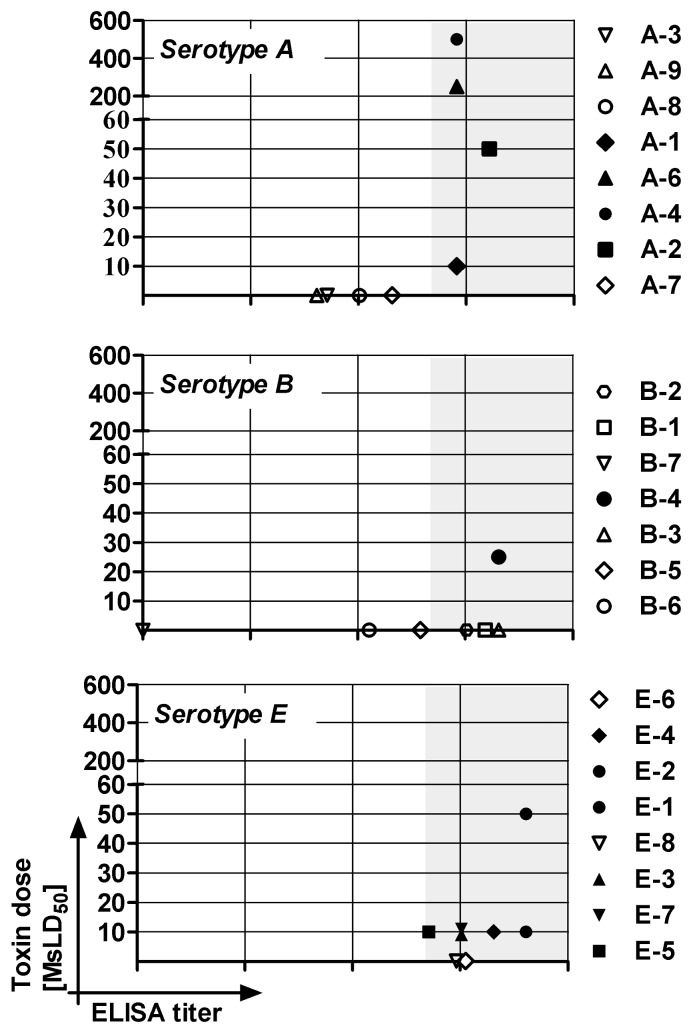
Individual neutralizing activity. A constant dilution of individual MAb ascitesfluids (1∶100) was pre-incubated with different toxin doses as described in materials and methods and then injected into mice. The results indicate the maximal toxin dose that each MAb could neutralize with respect to its anti-toxin ELISA titer. The MAb was considered neutralizing if 100% survival was achieved.

Immunization with Hc fragment, which contains most of the BoNT neutralizing epitopes, may favor the generation of neutralizing MAbs [7]. To test whether our native BoNT binding MAbs present neutralizing activity, different doses of serotype A, B, or E toxin were incubated with the relevant ascites fluids. The mixtures were then injected into mice, and survival was monitored (Figure 3). Four of nine type A specific MAbs protected mice against 10 MsLD_50_ of BoNT/A. Of the remaining MAbs, two (A-3 and A-7) were able to delay time to death (TTD) for 24 hours. The neutralizing MAbs were further tested for their potency against higher doses of BoNT/A. MAbs A-2, A-6 and A-4 neutralized doses of 50, 250 and 500 MsLD_50_, respectively. In anti-serotype B group, MAb B-4 was the only neutralizing MAb that protected against a toxin dose of 25 MsLD_50_. However, five out of the six remaining MAbs delayed TTD by 24 hours when tested against 10 MsLD_50_. In the anti-serotype E MAb panel, six out of eight MAbs protected mice from a 10 MsLD_50_ challenge, while the two remaining MAbs delayed TTD for 24 hours. The only MAb that presented increased anti-E neutralizing activity was E-1, which protected mice from a 50 MsLD_50_ dose of toxin E. Thus, neutralizing MAbs were obtained for each BoNT serotype. When the neutralization potency of each MAb was plotted against its anti-toxin ELISA titer, it was found that protecting MAbs were those that presented the highest ELISA titers (Figure 3). Results and rank of potency were maintained when neutralizing potency was normalized to IgG concentration of each MAb ascites sample.

### Neutralizing Activity of Oligoclonal Antibody Combinations

Recent studies have shown that a combination of several anti-BoNT MAbs may present synergistic neutralizing activity [29], [30]. Thus, the protective properties of a combined MAb preparation may potentially exceed the calculated additive effect of the mixture’s components (an algebraic summation of all individual neutralizing activities). We tested whether this phenomenon could also be demonstrated using the MAbs generated in our study. To restrict the total MAb combinations tested in mice, a rationale for combining MAbs in each serotypic group was developed. A mixture of distinct epitope binding MAbs has greater potential for synergism. Therefore, we conducted a competitive s-ELISA to categorize the MAbs according to their epitope recognition pattern.

A complete analysis of all possible MAb combinations was conducted, and the results are summarized in Table 4. The representative data from individual MAb assays are presented in Figure S3. The results showed that for each toxin serotype, two main epitopes could be resolved. One epitope was recognized by most of the Mabs, whereas the other epitope could only be bound by 1–2 MAbs.

**Table 4:** Epitope recognition analysis.

Serotype	Epitope Recognition group^a^	MAb
A	A1	A-1, A-2, A-3, A-4, A-6
	A2	A-8, A-9
B	B1	B-1, B-2, B-3, B-5, B-6
	B2	B-4
E	E1	E-2, E-3, E-4, E-5, E-6, E-7, E-8
	E2	E-1

a Epitope recognition groups within each serotype-specific panel of MAbs were determined by assessing all possible combinations of MAb pairs based on competition s-ELISA, as described in methods.

The *in vitro* competition results together with the supportive individual MAb neutralization data were used to assign several combinations of MAbs that may potentially present synergistic protective properties. The ascites fluid for each Mab was equally diluted and mixed with all of its other serotype-specific counterparts to form an oligoclonal cocktail. The MAb mixture was incubated with the homologous toxin and injected into three mice to monitor survival.

**Figure 4:**
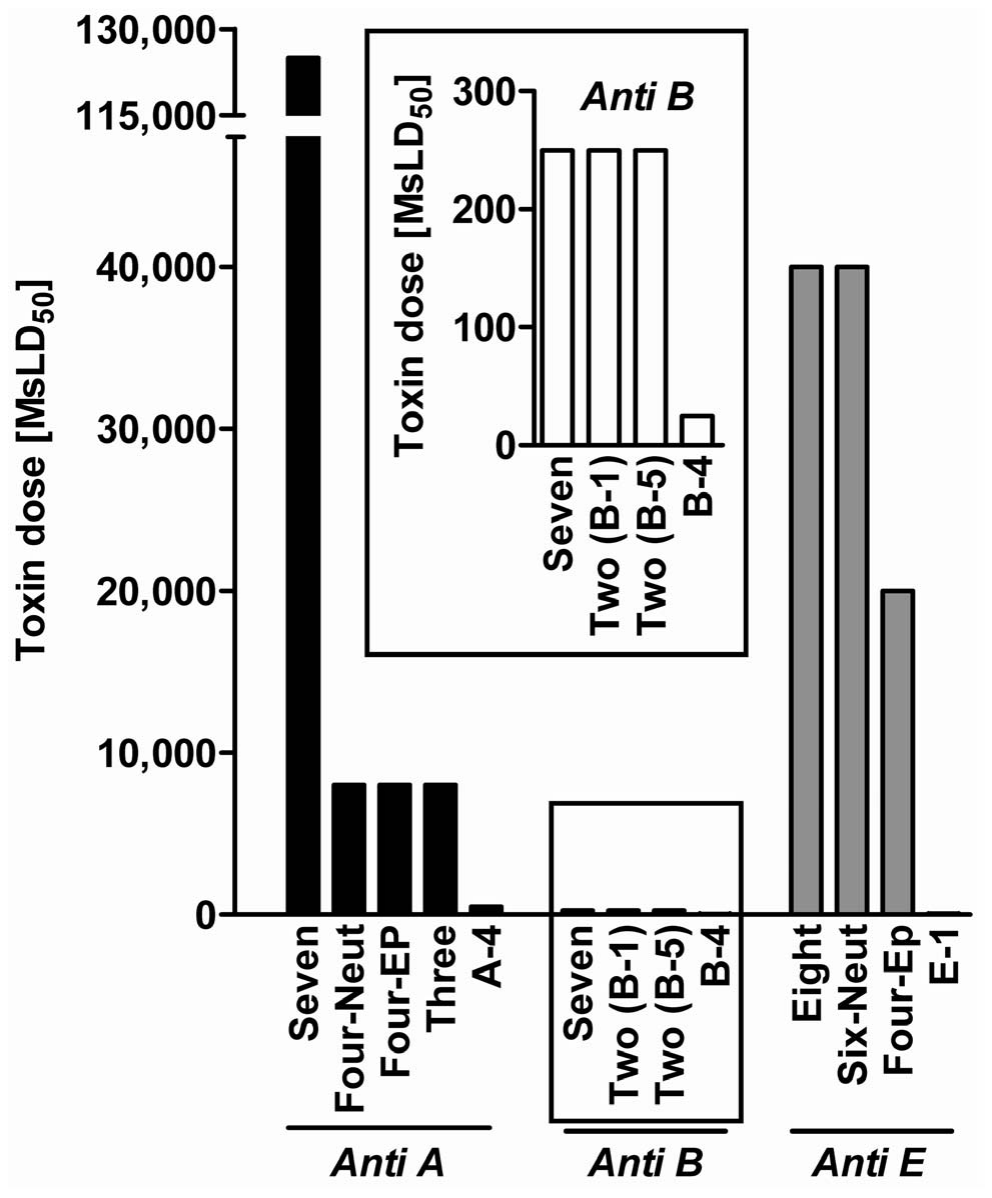
Neutralizing activity of oligoclonal combinations. Different toxin doses were pre-incubated with combinations of equally diluted MAb ascites fluids (final dilution 1∶200) and then injected to mice. The results indicate the maximal toxin dose that mice could withstand. Anti-serotype B MAb results are zoomed separately. Anti-serotype A MAb panel: ***Seven*** – [A-4, A-1, A-6, A-2, A-3, A-8, A-7]; ***Four-EP*** – [epitope recognition based MAbs A-4, A-1, A-3, A-8]; ***Four-Neut*** – [neutralizing MAbs A-4, A-1, A-6, A-2]; ***Three*** – [A-4, A-1, A-8 or A-3]. Anti-serotype B MAb panel: ***Seven*** – [B-4, B-2, B-1, B-3, B-6, B-5, B-7]; ***Two (B-1)*** – [B-4, B-1]; ***Two (B-5)*** – [B-4, B-5]. Anti-serotype E MAb panel: ***Eight*** – [E-2, E-3, E-4, E-5, E-6, E-7, E-8, E-1]; ***Six-Neut*** – [neutralizing MAbs E-2, E-3, E-4, E-5, E-7, E-1]; ***Four-EP*** – [epitope recognition based MAbs E-2, E-3, E-8, E-1].

The cocktail of seven anti-A MAbs neutralized 125,000 MsLD_50_ of toxin A (Figure 4)–a 154-fold improvement over the calculated additive effect of the mixture’s components (neutralization of 810 MsLD_50_). This result demonstrates a significant synergistic protective activity for the combined preparation. An even more pronounced synergistic neutralizing effect, a 400-fold improvement, was observed in the anti-E MAb combination. The mixture of all eight MAbs protected mice against a 40,000 MsLD_50_ of BoNT/E, whereas calculating the additive potency of the individual MAbs resulted in an expected neutralization of only 100 MsLD_50_. A combination of all seven anti-B MAbs presented a 10-fold synergistic effect, neutralizing 250 MsLD_50_ compared to the expected 25 MsLD_50_ (Figure 4).

Other combinations that included fewer MAbs based on either the best neutralizing MAbs or those representing different epitope recognition groups also improved neutralizing activity. Combining only the anti-serotype A MAbs that presented individual neutralizing activity (A-4, A-1, A-6, A-2) enabled protection against 8,000 MsLD_50_ (Figure 4, Four-Neut). Strikingly, the significantly reduced potency observed for the 4-clonal compared to the 7-clonal anti-BoNT/A cocktail (neutralization of 8,000 MsLD_50_ instead of 125,000 MsLD_50_) was attributed to the withdrawal of three non-neutralizing MAbs. The protective effect of the neutralizing 4-clonal cocktail was still 10-fold higher than the calculated additive effect of its components (neutralization of 810 MsLD_50_). When two of the neutralizing MAbs (A-2, A-6) in the 4-clonal cocktail were substituted with two non-neutralizing MAbs, one of each epitope recognition group (A-3 from epitope A1 and A-8 from epitope A2, Table 4), the new 4-clonal cocktail maintained neutralizing activity and protected mice against 8,000 MsLD_50_ of BoNT/A (Figure 4, Four-Ep). Similar protection could even be demonstrated using a 3-clonal cocktail (A-4, A-1 A-8/A-3). However, attempts to use 2-clonal cocktails did not result in synergism.

The same rationale was used to study the protective effect of anti-serotype E MAb combinations. In contrast to anti-A cocktails, omitting the two non-neutralizing MAbs from the 8-clonal combination did not impair potency, allowing mice to be protected from a 40,000 MsLD_50_. Further reduction of the MAb number to create a 4-clonal cocktail based on the epitope recognition pattern led to only a two-fold decrease in potency (neutralizing 20,000 MsLD_50_).

The individual neutralization assays showed that in the serotype B-specific panel, B-4 was the only neutralizing MAb (protecting against 25 MsLD_50_). It was also determined that B-4 binds a separate epitope from all other non-neutralizing MAbs. A combination of B-4 with only one non-neutralizing MAb (B-5 or B-1) was sufficient to induce synergism and protect mice against 250 MsLD_50_ of BoNT/B.

The high neutralizing activity obtained for the MAb cocktails in the current study encouraged us to measure their potency using the same method employed to determine high-titer pharmaceutical anti-botulism products [28]. In each serotype-specific group, the cocktail that protected mice against the highest toxin dose was tested. The 7-clonal anti-serotype A cocktail had a neutralizing antibody concentration of 600 IU/ml, whereas a concentration 1,330 IU/ml was measured for the 8-clonal anti-serotype E mixture. These values are comparable to the antibody neutralizing concentrations in pharmaceutical polyclonal anti-botulism preparations [31].

## Discussion

Botulism is a neuroparalytic syndrome that can progress to respiratory malfunction and death. To successfully control the disease, rapid diagnosis and treatment are essential. Both of these clinical challenges are currently being addressed by anti-botulinum polyclonal antibody preparations [14], [31].

However, polyclonal antibodies may have several limitations that can be potentially overcome by monoclonal antibodies. Both high affinity and highly specific reagents are required in order to differentially detect relevant concentration of BoNT serotypes in the blood. Anti-BoNT MAbs have been integrated in highly specific and sensitive immuno-diagnostic assays [14]. With regard to treatment, current pharmaceutical preparations are derived from hyperimmune equine sera. Therefore, these treatments are associated with significant side effects, including hypersensitivity reactions such as serum sickness and anaphylaxis [12], [13]. Human-derived or humanized MAb-based preparations should have a substantially decreased incidence of side effects and improved pharmacokinetics [15], [22].

To simultaneously generate MAbs against botulinum serotype A, B, and E, we conducted a trivalent mice immunization with a mixture of the recombinant Hc of each of the three neurotoxins. We then followed the immunization with parallel differential robotic hybridoma screening. For optimal specificity and neutralization potency, we chose to use a recombinant Hc fragment as the immunogen. Hc is the domain presenting the lowest homology between different botulinum serotypes [6] and contains most of the toxin neutralizing epitopes [7], [10]. Multiplex immunization for MAb production minimizes the number of animals required for immunization and the overall tissue culture load related to hybridoma generation [15]. The simultaneous injection of more than one antigen into a single mouse has already proven successful [32]. One concern regarding the use of trivalent immunization was the formation of a biased immune response against one of the BoNT serotypes. This phenomenon manifest as a reduced anti-BoNT titer compared to a monovalent immunization with the same antigen. We found that mice immunized with HcA alone or with HcA as part of the trivalent mixture had similar anti-BoNT/A titers. During the immunization process, a reduced anti-BoNT/E titer developed. The lower immunogenicity of HcE compared to HcA and HcB was previously demonstrated [8], [9], [33]. To overcome the low HcE titer, we adjusted the immunization protocol by adding mono-HcE injections (Figure 1A). The comparable anti-BoNT titers and number of MAbs obtained against each of the three BoNT serotypes confirmed the ability to reach balanced immunity to the three antigens. Similar results were obtained by Chiarella and his colleagues, who compared single and multiplex immunization strategies and showed that the latter was the most effective [34].

A major challenge in the production of MAbs using the multiplex immunization strategy is that it is necessary to conduct simultaneous screening of hybridoma supernatants against the different antigens. Several approaches have been used to address this challenge, including antigen microarrays and fluorometric microvolume assays [15], [32], [35]. We chose to conduct a simultaneous high-throughput robotic screening that enabled us to analyze ∼1300 hybridoma supernatants in three parallel ELISAs for serotypes A, B, and E. This approach allowed 42 ELISA plates to be screened per day. Low capture antigen concentrations were used to increase the assay stringency. The capture antigens were complex toxoids that, according to their molecular weights [36], comprised only ∼5.5%, ∼10%, and ∼17% of the Hc portion in each A, B, and E toxoid, respectively. Thus, the actual amounts of relevant capture antigen were only ∼25–75 ng protein per well, which favors the selection of high-affinity MAbs. Indeed, most of our MAbs presented very high titers, and several exceeded the titers of anti-complex and anti-Hc PAbs. Seven to nine MAbs specific to each of the serotypes A, B, and E were obtained in the current study. This yield is comparable to anti-toxin MAbs in other studies [21], suggesting that the high stringency did not hinder the efficiency of the selection process.

Despite dramatic variation within residues, sequence homology among Hc fragments of BoNT serotypes A, B and E still exists [6]. Presentation in excess of such common sequences in the trivalent vaccine preparation could potentially lead to the generation of cross-reactive MAbs. However, only one out of 25 hybridoma clones was found to be bi-serotype specific. This result suggests that common epitopes among all three serotypes have reduced immunogenicity. Cross-reactive MAbs might not be advantageous for diagnostics but may be favorable for therapeutic use. In an effort to produce cross-reactive MAbs to A, B, and E serotypes selectively, Corbett et al. used a different sequential immunization strategy. This strategy used mice initially immunized with HcA and boosted with HcB and HcE antigens [35]. The result was 11 MAbs, of which two were cross-reactive to all three Hc antigens and one MAb was cross-reactive to HcA and HcE. In another work, it was found that out of 35 yeast-displayed single-chain variable fragment (scFv) tested, only one bound BoNT A and B and another one bound BoNT A, B, E, and F [37]. The data from these studies are in line with our results, which demonstrate that cloning of multi-serotype specific anti-BoNT MAbs is challenging.

Anti-Hc PAbs present increased specificity but reduced anti-botulinum complex ELISA titers compared to their anti-complex counterparts (Tables 1, 2, 3). The elevated specificity results from the relative low homology of the Hc domain among different BoNT serotypes [6]. Conversely, the reduced titer stems from the small proportion of Hc (5–17%) in the toxin complex [36]. In the current study, we were able to select MAbs with both high specificity and high ELISA titer. The specificity of some anti-serotype A, B and E MAbs was found to be three orders of magnitude higher than the already specific anti-Hc PAbs. Furthermore, these MAbs were up to five orders of magnitude more specific than the anti-complex PAbs although maintaining similar or even increased anti-botulinum ELISA titers. Normalizing the titers to IgG concentration had no significant effect on the results or the interpretation drawn (Table S1).

Because the production of MAbs in the current study was based on immunization with recombinant Hc fragments and screening against toxoid, it was necessary to test the MAbs’ ability to bind native botulinum toxin. The s-ELISA results showed that almost all of the MAbs bound to their native toxins, with 89% (8/9), 100% (7/7), and 100% (8/8) recognition for anti-A, B, and E serotypes, respectively. In two different studies where both the immunization and screening were conducted using recombinant Hc fragments, it was found that 41% and 74% of the Hc specific MAbs were able to bind the native toxin [35], [38]. In another study, where immunization and screening were conducted using botulinum toxoid, a small percentage of native toxin binding MAbs was reported [19]. The relatively high frequency of native toxin recognizing clones in our work might be attributed to the selection strategy that combined two different non-toxic forms of the target toxin (Hc and toxoid) as an immunogen and a screening antigen.

The ability of Hc-specific MAbs to bind native toxin suggested that these MAbs might present protective properties. Indeed, we were able to obtain neutralizing MAbs within each serotype specific group. The majority of MAbs protected against a toxin dose of 10 MsLD_50_. Comparable potency of anti-BoNT MAbs has been demonstrated by others [38], [39], [40], [41]. The neutralizing potency observed in our study was associated with an increased anti-toxin ELISA titer, as all neutralizing MAbs exhibited a minimal ELISA titer of 5×10^6^. In accordance with these results, Maynard et al. showed that protection against anthrax toxin by recombinant Ab fragments correlated with antigen affinity [42]. Nevertheless, anti-BoNT MAbs with high ELISA titers but no neutralizing activity were also demonstrated in our study. Thus, anti-serotype B MAbs B-4 and B-3 had the highest anti-toxin ELISA titer (2×10^7^). However, while MAb B-4 protected mice from a 25 MsLD_50_ dose of BoNT/B, MAb B-3 lacked neutralizing activity. The same phenomenon was demonstrated in anti-serotype A and E MAbs. These findings suggest that high ELISA titers may be a prerequisite but may not serve as a predictive parameter for increased neutralizing potency.

The neutralization potency of anti-botulinum MAbs, including those found in the current study, is significantly lower than that of polyclonal-based approved pharmaceuticals [31]. Even our best MAb that individually neutralized 500 MsLD_50_ of BoNT/A (A-4) had a calculated potency of 10 IU/ml (according to the pharmacopeia definition that 1 IU neutralizes 10,000 MsLD_50_ of BoNT, [28]). This potency is far below that reported in equine plasma and for pharmaceutical antitoxins [43] and is the major obstacle in applying MAb-based anti-botulinum therapy. Another drawback of using an individual MAb for anti-botulinum therapy is related to the existence of several subtypes within the toxin serotypes. Thus, a neutralizing epitope expressed on a specific subtype might be modified or even absent on another, leading to a significant decrease in the efficacy of the relevant neutralizing MAb [44], [45], [46]. Both the challenges of the low neutralizing titer of single MAbs and the unexpected efficacy owing to sequence variation among different subtypes can be addressed by a preparation based on the combination of several MAbs.

To design optimal oligoclonal combinations that consist of hetero-epitope binding MAbs, we first analyzed the epitope recognition pattern using a competitive s-ELISA [46]. This preliminary *in vitro* analysis enabled us to resolve two main epitopes for each BoNT serotype. These findings are in agreement with those of others. Using immunometric assays that conceptually resembled our competitive s-ELISA, Volland et al. analyzed 14 anti-serotype A MAbs and found five distinct epitopes on the HcA fragment [47]. In another study, Pless et al. epitope-mapped 11 anti-A neutralizing MAbs using SPR and showed two distinct protective non-overlapping epitopes [38].

Further sequencing data may be required in order to finally determine whether individual MAbs sharing the same isotype and epitope recognition group are unique. Nevertheless, the *in vitro* epitope binding resolution in our study was in accordance with the individual neutralization results. All Anti-serotype A neutralizing MAbs bound a similar epitope in the *in vitro* competition assay. Only one MAb of this epitope binding group (A-3) did not present neutralizing activity, most likely due to a significantly reduced anti-toxin ELISA titer. In type B specific MAbs, B-4 was the only antibody that presented significant neutralizing activity and was determined to bind a separate epitope from all other type B specific MAbs. The same result was apparent in type E specific Mabs, where MAbs E-6 and E-8 were the only MAbs that did not neutralize toxin and were sub-grouped together according to s-ELISA analysis. Thus, the neutralization results could at least partially validate our epitope recognition analysis.

Oligoclonal combinations consisting of all MAbs in each serotype-specific group were prepared and tested for their neutralizing activity. Additional combinations comprising fewer MAbs according to the epitope recognition categorization were also tested. We observed high synergistic neutralizing activity (up to 400-fold) in oligoclonal cocktails. The synergy of MAb cocktails has been previously demonstrated by others in the cases of tetanus toxin, HIV, and anthrax [48], [49], [50]. Oligoclonal MAb combinations have also proven to be highly synergistic for BoNT therapy, as three different antitoxin drugs based on either anti-BoNT A, B, or E MAb combinations have been developed recently [13], [22].

Interestingly, MAbs that failed to present individual neutralizing activity in our study made a significant contribution to the synergistic effect observed in the oligoclonal preparation. The significantly reduced potency observed for the 4-clonal compared to the 7-clonal anti-BoNT/A cocktail (neutralization of 8,000 MsLD_50_ instead of 125,000 MsLD_50_) was due to the withdrawal of three non-neutralizing MAbs. Substituting two neutralizing MAbs with two non-neutralizing Mabs, or even reducing the number of MAbs to form a 3-clonal cocktail, maintained the same neutralizing activity. A reduced number of MAbs may be advantageous for pharmaceutical purposes [51]. Alternatively, a combination of the only neutralizing anti-BoNT/B MAb (B-4) with a single non-neutralizing MAb (B-5) was sufficient to achieve a 10-fold synergistic effect, neutralizing 250 MsLD_50_ instead of 25 MsLD_50_. Additionally, replacing B-5 with another non-neutralizing MAb (B-1) maintained the same protective effect. This result confirms that adding a single non-neutralizing MAb to the neutralizing antibody induced synergism. The induction of synergistic effects due to the combination of non-neutralizing MAbs could be explained by several mechanisms. First, it is expected that the functional affinity of the mixture of MAbs is greatly increased, as has been demonstrated by others [30]. Second, the binding of antibodies to multiple sites on the toxin might cause a steric hindrance effect, thus preventing the toxin from attaching or entering their target cells [29]. Third, multimeric antibody decoration of a target antigen is considered necessary to permit binding to low affinity Fc receptor and mediating clearance from serum [52], [53]. This Fc-mediated clearance mechanism was recently used to demonstrate a novel strategy for the development of recombinant antitoxin therapeutics [54].

The highly protective 7-clonal anti-A and 8-clonal anti-E MAb combinations were tested for their neutralization potency in IU/ml according to the pharmacopeia assay. The potency of these two combinations could successfully compete with horse derived PAb pharmaceutical preparations [31]. Humanization of the corresponding MAbs may result in oligoclonal antibody preparations that should prove much safer and more adequate for pharmaceutical use than the current horse PAb-based drug. These advantageous characteristics together with the potential for scaling up MAb production should enable the generation of unlimited quantities of anti-BoNT with reduced side effects.

To conclude, our study provides quantitative insight into the synergistic protective effect that ensues when individual MAbs are combined to an oligoclonal antibody preparation; a phenomenon with potent implications in the field of antibody therapy. Moreover, the study provides new data regarding the individual contribution of neutralizing, and more importantly, of non-neutralizing MAbs to the synergistic protective effect.

## Supporting Information

Figure S1 **Schematic representation of the screening process.** Splenic cells from mice hyperimmune to HcA, HcB, and HcE were fused with mouse myeloma cells to generate hybridomas. Then, 150 µl of the supernatants from each well of the hybridoma cell culture plate were distributed to three different ELISA plates, 50 µl per well, and tested simultaneously in three parallel ELISA assays (for each of the serotypes A, B, and E) using high throughput robotic systems (GENESIS RMP 200/150, TECAN).
(TIF)

Figure S2 **Specificity of MAbs.** Serotype-specific titers of MAb and control PAbs were determined with ELISA. Plates were coated with toxoids A, B, or E, and each antibody was simultaneously tested against all three serotypes. ELISA was performed as described in the methods section. Homologous titers and heterologous cross-titers were determined as the last dilution with O.D. greater than three standard deviations above mean background. αHc = mouse anti-Hc; αComp = rabbit anti-toxin complex.
(TIF)

Figure S3 **Assessment of epitope recognition groups.** Competitive s-ELISA was performed for each serotype specific group of MAbs. Native A, B, or E toxins were captured by anti-complex PAbs. Serial dilutions of purified unlabeled MAbs were mixed with a constant concentration of each of the biotin-labeled MAb and incubated with the relevant toxin. Enzyme-conjugated streptavidin (SA) was used as the reporting agent for the presence of biotin-labeled MAbs. Maximum O.D. was determined without inhibition of purified MAb and was normalized to be 100% reaction of the reporting agent. Data for two representative MAbs from each serotype-specific group are depicted (anti-A upper panels, anti-B mid panels and anti-E lower panels). Anti-E MAb E-1 could not be biotin labeled and was therefore tested as a competitor only. It was unable to out compete any of the other anti-E MAbs, which suggests that it could recognize a distinct epitope. Anti-A MAb A-7 did not provide a conclusive result.
(TIF)

Table S1 **IgG concentration in Ascites fluids used in the study.**
(DOCX)
