# Supplementary figures and images for: Evaluating the Synergistic Neutralizing Effect of Anti-Botulinum Oligoclonal Antibody Preparations
Source: PLoS One. 2014 Jan 27;9(1):e87089. doi: 10.1371/journal.pone.0087089 (PMC3903612; doi:10.1371/journal.pone.0087089)

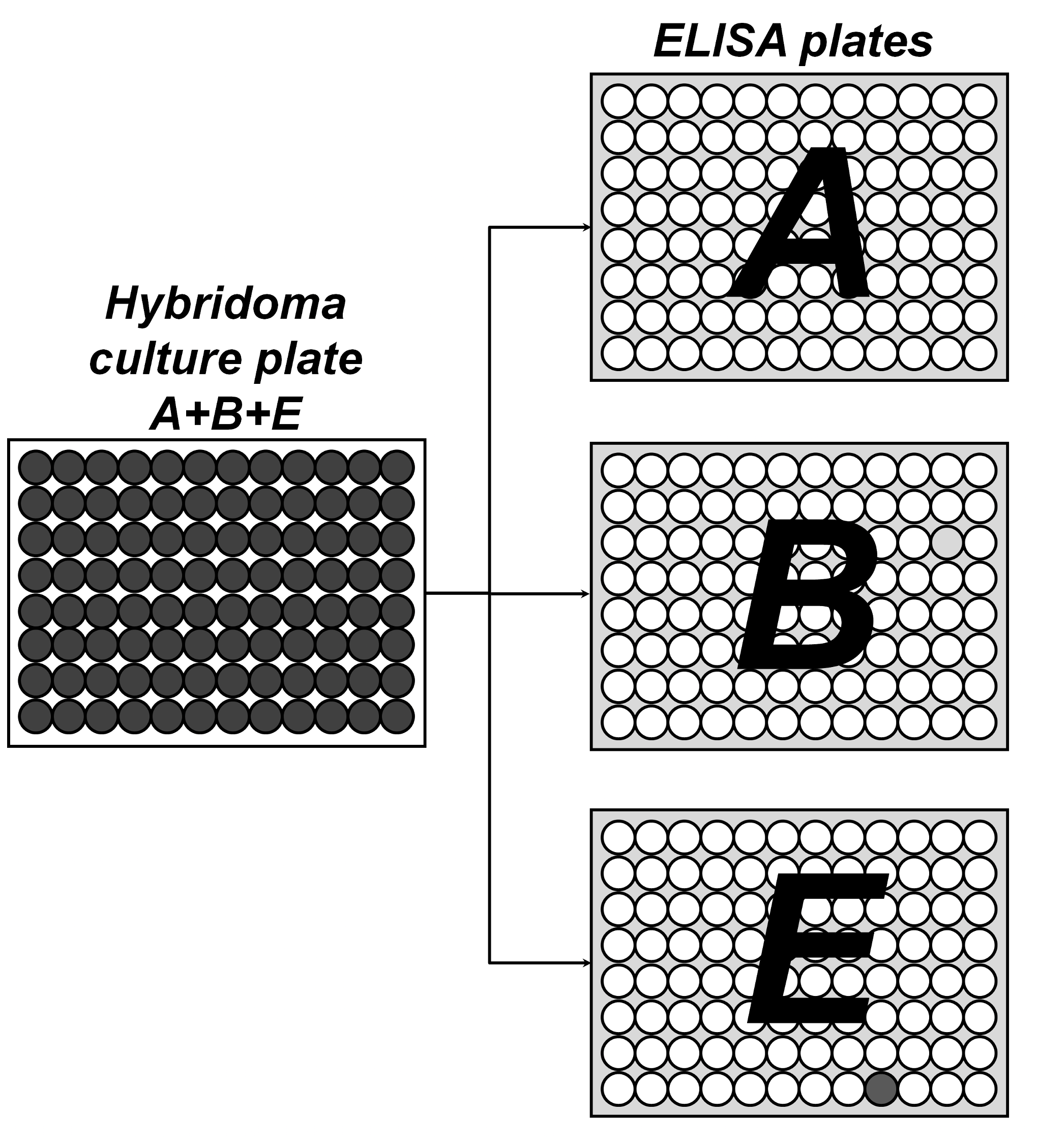

Supplement: Figure S1 — Schematic representation of the screening process. Splenic cells from mice hyperimmune to HcA, HcB, and HcE were fused with mouse myeloma cells to generate hybridomas. Then, 150 µl of the supernatants from each well of the hybridoma cell culture plate were distributed to three different ELISA plates, 50 µl per well, and tested simultaneously in three parallel ELISA assays (for each of the serotypes A, B, and E) using high throughput robotic systems (GENESIS RMP 200/150, TECAN). (TIF) [file pone.0087089.s001.tif]

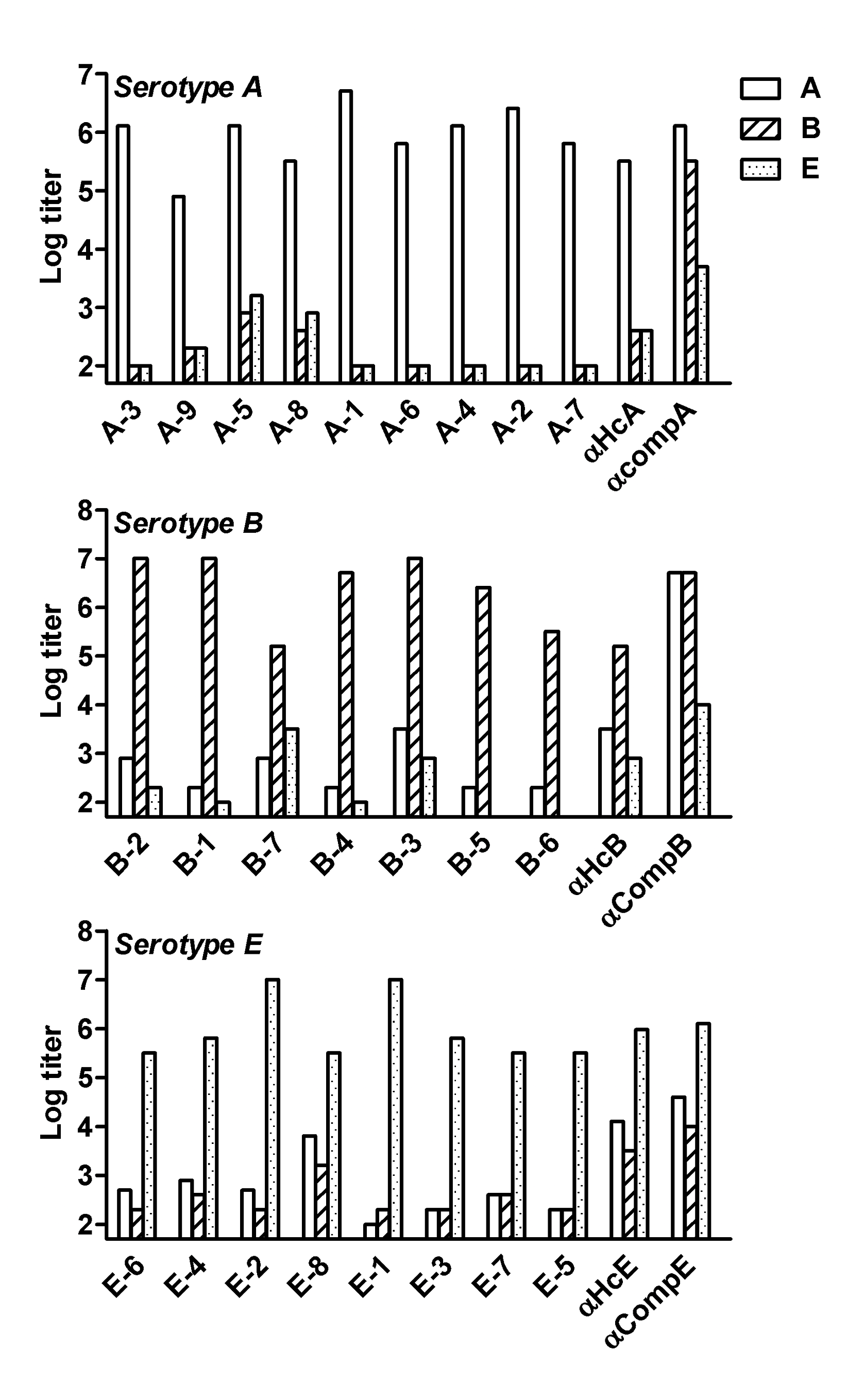

Supplement: Figure S2 — Specificity of MAbs. Serotype-specific titers of MAb and control PAbs were determined with ELISA. Plates were coated with toxoids A, B, or E, and each antibody was simultaneously tested against all three serotypes. ELISA was performed as described in the methods section. Homologous titers and heterologous cross-titers were determined as the last dilution with O.D. greater than three standard deviations above mean background. αHc = mouse anti-Hc; αComp = rabbit anti-toxin complex. (TIF) [file pone.0087089.s002.tif]

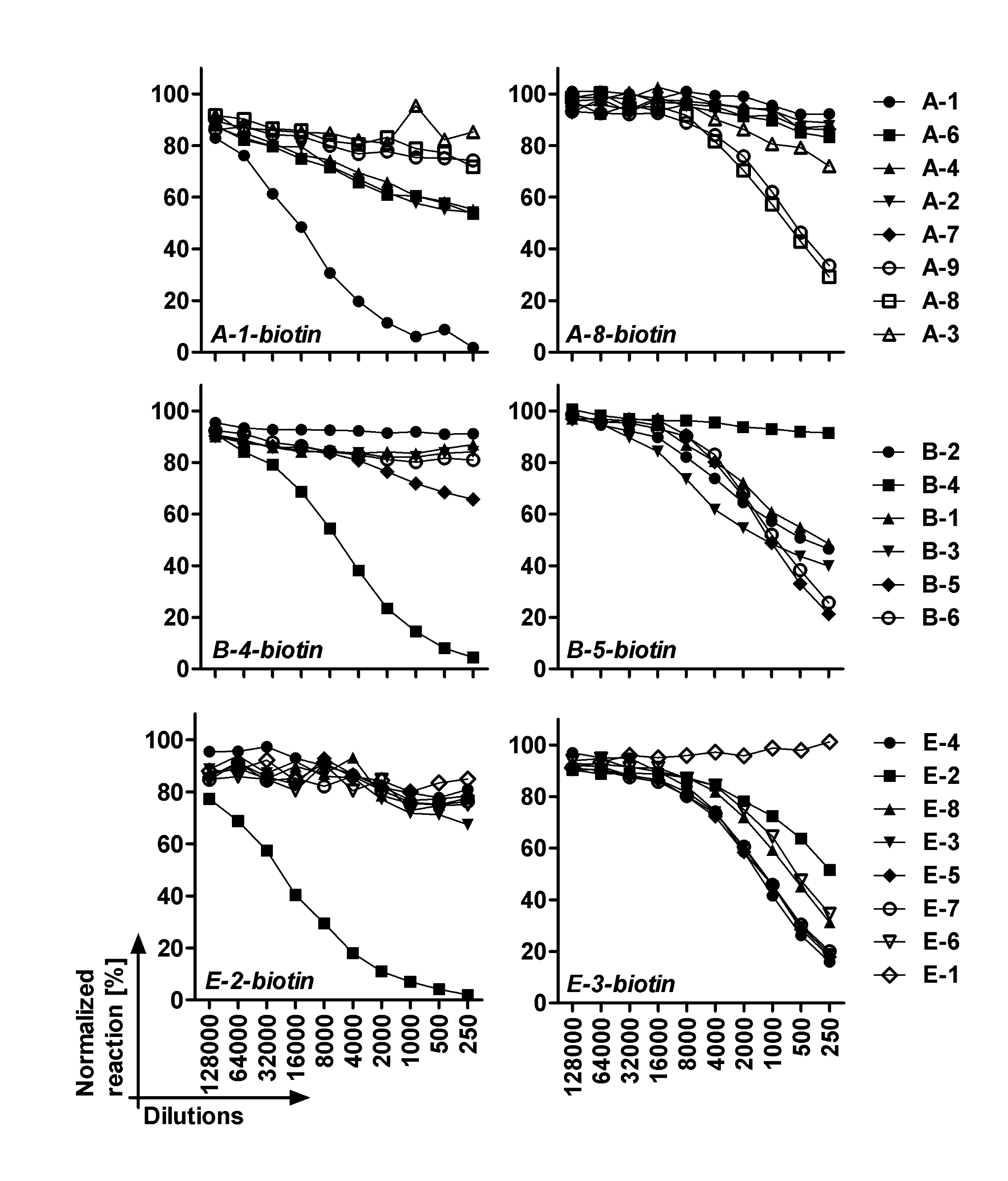

Supplement: Figure S3 — Assessment of epitope recognition groups. Competitive s-ELISA was performed for each serotype specific group of MAbs. Native A, B, or E toxins were captured by anti-complex PAbs. Serial dilutions of purified unlabeled MAbs were mixed with a constant concentration of each of the biotin-labeled MAb and incubated with the relevant toxin. Enzyme-conjugated streptavidin (SA) was used as the reporting agent for the presence of biotin-labeled MAbs. Maximum O.D. was determined without inhibition of purified MAb and was normalized to be 100% reaction of the reporting agent. Data for two representative MAbs from each serotype-specific group are depicted (anti-A upper panels, anti-B mid panels and anti-E lower panels). Anti-E MAb E-1 could not be biotin labeled and was therefore tested as a competitor only. It was unable to out compete any of the other anti-E MAbs, which suggests that it could recognize a distinct epitope. Anti-A MAb A-7 did not provide a conclusive result. (TIF) [file pone.0087089.s003.tif]
