# Supplementary material for: Evaluating the Synergistic Neutralizing Effect of Anti-Botulinum Oligoclonal Antibody Preparations
Source: PLoS One. 2014 Jan 27;9(1):e87089. doi: 10.1371/journal.pone.0087089 (PMC3903612; doi:10.1371/journal.pone.0087089)
Supplement: Table S1 — IgG concentration in Ascites fluids used in the study. (DOCX) [file pone.0087089.s004.docx]

**Table S1: IgG concentration in Ascites fluids used in the study**

| Anti BoNT A MAbs | Average IgG^a^ (mg/ml) | Standard deviation (STD) |
| --- | --- | --- |
| A-1 | 5.55 | 0.68 |
| A-2 | 6.56 | 1.06 |
| A-3 | 1.11 | 0.13 |
| A-4 | 4.67 | 0.67 |
| A-5 | 3.10 | 0.39 |
| A-6 | 7.63 | 1.03 |
| A-7 | 3.52 | 0.99 |
| A-8 | 7.32 | 1.32 |
| A-9 | 2.58 | 0.39 |
| **Average Anti-A MAbs** | **4.67** | **2.26** |
|  |  |  |
| **Anti BoNT B MAbs** |  |  |
| B-1 | 4.54 | 0.47 |
| B-2 | 5.36 | 0.58 |
| B-3 | 3.44 | 0.56 |
| B-4 | 2.75 | 0.25 |
| B-5 | 1.58 | 0.27 |
| B-6 | 3.42 | 1.01 |
| B-7 | 2.06 | 0.11 |
| **Average Anti-B MAbs** | **3.31** | **1.23** |
|  |  |  |
| **Anti BoNT E MAbs** |  |  |
| E-1 | 3.82 | 0.26 |
| E-2 | 3.93 | 0.28 |
| E-3 | 4.44 | 0.29 |
| E-4 | 8.39 | 1.08 |
| E-5 | 5.94 | 0.78 |
| E-6 | 4.27 | 0.27 |
| E-7 | 4.75 | 0.39 |
| E-8 | 4.55 | 0.32 |
| **Average Anti-E MAbs** | **5.01** | **1.42** |
|  |  |  |
| **Average all MAbs** | **4.39** | **1.86** |

^a^IgG concentration were determined by a specific ELISA as described in the updated Method section. Samples were tested in triplicates.
